# Long-Term Symptoms Among Adults Tested for SARS-CoV-2 — United States, January 2020–April 2021

**DOI:** 10.15585/mmwr.mm7036a1

**Published:** 2021-09-10

**Authors:** Valentine Wanga, Jennifer R. Chevinsky, Lina V. Dimitrov, Megan E. Gerdes, Geoffrey P. Whitfield, Robert A. Bonacci, Miriam A.M. Nji, Alfonso C. Hernandez-Romieu, Jessica S. Rogers-Brown, Tim McLeod, Julie Rushmore, Caitlyn Lutfy, Dena Bushman, Emilia Koumans, Sharon Saydah, Alyson B. Goodman, Sallyann M. Coleman King, Brendan R. Jackson, Jennifer R. Cope

**Affiliations:** ^1^CDC COVID-19 Response Team; ^2^Epidemic Intelligence Service, CDC; ^3^Oak Ridge Institute for Science and Education, Oak Ridge, Tennessee; ^4^National Center for Chronic Disease Prevention and Health Promotion, CDC.

Long-term symptoms often associated with COVID-19 (post-COVID conditions or long COVID) are an emerging public health concern that is not well understood. Prevalence of post-COVID conditions has been reported among persons who have had COVID-19 (range = 5%–80%), with differences possibly related to different study populations, case definitions, and data sources (*1*). Few studies of post-COVID conditions have comparisons with the general population of adults with negative test results for SARS-CoV-2, the virus that causes COVID-19, limiting ability to assess background symptom prevalence (*1*). CDC used a nonprobability-based Internet panel established by Porter Novelli Public Services* to administer a survey to a nationwide sample of U.S. adults aged ≥18 years to compare the prevalence of long-term symptoms (those lasting >4 weeks since onset) among persons who self-reported ever receiving a positive SARS-CoV-2 test result with the prevalence of similar symptoms among persons who reported always receiving a negative test result. The weighted prevalence of ever testing positive for SARS-CoV-2 was 22.2% (95% confidence interval [CI] = 20.6%–23.8%). Approximately two thirds of respondents who had received a positive test result experienced long-term symptoms often associated with SARS-CoV-2 infection. Compared with respondents who received a negative test result, those who received a positive test result reported a significantly higher prevalence of any long-term symptom (65.9% versus 42.9%), fatigue (22.5% versus 12.0%), change in sense of smell or taste (17.3% versus 1.7%), shortness of breath (15.5% versus 5.2%), cough (14.5% versus 4.9%), headache (13.8% versus 9.9%), and persistence (>4 weeks) of at least one initially occurring symptom (76.2% versus 69.6%). Compared with respondents who received a negative test result, a larger proportion of those who received a positive test result reported believing that receiving a COVID-19 vaccine made their long-term symptoms better (28.7% versus 15.7%). Efforts to address post-COVID conditions should include helping health care professionals recognize the most common post-COVID conditions and optimize care for patients with persisting symptoms, including messaging on potential benefits of COVID-19 vaccination.

During April 9–23, 2021, Porter Novelli Public Services and ENGINE Insights^†^ conducted a nonprobability-based Internet panel survey among 6,021 noninstitutionalized U.S. adults aged ≥18 years via the Lucid platform.^§^ Quota sampling and statistical weighting were used to align the sample with U.S. population distributions by sex, age group, U.S. Census region, race and ethnicity, and education. Respondents self-reported ever having received a positive SARS-CoV-2 test result (698), always receiving a negative test result (2,437), or never having been tested for SARS-CoV-2 (2,750); only deidentified respondents who reported having received either a positive or a negative test result were included in this analysis. Respondents who received a negative test were selected to establish the prevalence of post-COVID symptoms in a population that did not receive a COVID-19 diagnosis. Assessment of initial symptoms, including symptoms that might have commenced before testing,^¶^ was conducted by asking respondents who received a positive test result, “During the month of your first positive COVID-19 test, which, if any, of the following symptoms did you experience?” followed by a list of symptoms. Respondents who received a negative test result were asked, “Since January 2020, which, if any, of the following symptoms have you experienced?” Long-term symptoms were assessed by asking those who received a positive test result, “Which, if any, of your symptoms lasted longer than 4 weeks after your first positive COVID-19 test?”; those who received a negative test result were asked, “Which, if any, of your symptoms lasted longer than 4 weeks since you first experienced the symptoms?” Respondents were asked about health care use and receipt of ≥1 dose of a COVID-19 vaccine. Vaccine impact related to symptoms was assessed by asking respondents** how receiving a COVID-19 vaccination affected their long-term symptoms (those lasting ≥4 weeks).^††^

Point estimates and 95% CIs were calculated, overall and by demographic characteristics (age group, sex, marital status, highest educational attainment, employment, 2020 household income, race and ethnicity, U.S. Census region, and community type^§§^). Comparisons of demographic characteristics and symptoms were performed using chi-square tests; p-values <0.05 were considered statistically significant. All analyses were conducted using SAS (version 9.4; SAS Institute) and were weighted by sex, age group, region, race and ethnicity, and education. This activity was reviewed by CDC and was conducted consistent with applicable federal law and CDC policy.^¶¶^

Among the 3,135 adults who reported having been tested for SARS-CoV-2 since January 2020, the weighted prevalence of ever receiving a positive test result was 22.2% (Table 1). Compared with respondents who received a negative test result (2,437), those who received a positive test result (698) were younger (median age = 39.3 years versus 45.3 years), and a higher proportion were working (70.5% versus 61.6%), had higher household income (50.8% versus 43.9% made ≥$60,000), and lived in an urban community (43.8% versus 37.6%).

**TABLE 1:** Demographic characteristics of respondents aged ≥18 years who received at least one positive SARS-CoV-2 test result or only negative SARS-CoV-2 test results since January 2020 (N = 3,135), by selected characteristics — Porter Novelli Internet survey, United States, April 2021

Characteristic	Testing status, weighted % (95% CI)		p-value^†^
	At least one positive SARS-CoV-2 test result (n = 698)*	All negative SARS-CoV-2 test results (n = 2,437)*	
**Overall**	22.2 (20.6–23.8)	77.8 (76.2–79.4)	—
**Age group, yrs**			
18–29	26.3 (22.8–29.7)	23.2 (21.4–24.9)	<0.001
30–39	25.4 (22.0–28.8)	19.0 (17.4–20.6)	<0.001
40–49	18.6 (15.1–22.1)	16.4 (14.7–18.2)	<0.001
50–59	15.0 (11.8–18.2)	16.6 (14.8–18.4)	<0.001
60–69	10.3 (7.8–12.8)	16.4 (14.8–18.0)	<0.001
≥70	4.4 (2.8–6.0)	8.4 (7.2–9.5)	<0.001
**Sex**			
Male	51.5 (47.4–55.7)	48.5 (46.3–50.7)	0.204
Female	48.5 (44.3–52.6)	51.5 (49.3–53.7)	0.204
**Marital status**			
Married	54.0 (49.9–58.1)	49.2 (47.0–51.4)	0.197
Living with a partner	10.2 (7.7–12.6)	10.2 (8.8–11.6)	0.197
Single and never been married	22.2 (18.7–25.6)	24.4 (22.6–26.3)	0.197
Other**^§^**	13.6 (10.6–16.6)	16.1 (14.5–17.8)	0.197
**Highest level of education completed**			
Some high school or less	4.7 (2.8–6.6)	5.2 (4.1–6.3)	0.545
High school graduate/some college	50.7 (46.6–54.8)	49.2 (47.0–51.3)	0.545
2-year college/technical school	7.8 (5.7–10.0)	8.5 (7.3–9.8)	0.545
4-year college/some postgraduate education	19.6 (16.6–22.5)	21.7 (20.1–23.3)	0.545
Postgraduate degree	17.2 (14.4–19.9)	15.4 (14.1–16.8)	0.545
**Employment status** ^¶^			
Employed	70.5 (66.6–74.3)	61.6 (59.5–63.8)	<0.001
Unemployed	17.8 (14.5–21.1)	19.8 (17.9–21.6)	<0.001
Retired	11.7 (9.0–14.4)	18.6 (16.9–20.3)	<0.001
**Household income in 2020, USD**			
<25,000	14.5 (11.4–17.5)	20.4 (18.5–22.2)	0.016
25,000–49,999	28.2 (24.4–32.0)	27.8 (25.8–29.9)	0.016
50,000–74,999	17.2 (14.0–20.4)	17.3 (15.7–19.0)	0.016
75,000–99,999	12.0 (9.5–14.5)	11.3 (9.9–12.6)	0.016
100,000–149,999	16.3 (13.5–19.2)	14.4 (13.0–15.7)	0.016
≥150,000	11.8 (9.2–14.3)	8.9 (7.8–10.0)	0.016
**Race/Ethnicity**			
White, non-Hispanic	59.8 (55.5–64.0)	62.0 (59.7–64.2)	0.190
Black or African-American, non-Hispanic	12.4 (9.6–15.3)	12.3 (10.8–13.9)	0.190
Other,** non-Hispanic	6.2 (4.1–8.4)	8.2 (6.9–9.5)	0.190
Hispanic	21.6 (17.6–25.5)	17.5 (15.6–19.4)	0.190
**U.S. Census region^††^**			
Northeast	21.5 (18.1–24.9)	19.0 (17.3–20.6)	0.156
Midwest	21.1 (17.7–24.5)	19.5 (17.7–21.2)	0.156
South	38.1 (34.1–42.1)	38.4 (36.3–40.5)	0.156
West	19.3 (16.1–22.5)	23.1 (21.3–25.0)	0.156
**Community type**			
Urban	43.8 (39.8–47.9)	37.6 (35.5–39.7)	0.028
Suburban	39.8 (35.7–43.9)	44.1 (41.9–46.3)	0.028
Rural	16.4 (13.3–19.5)	18.3 (16.6–20.0)	0.028

**Abbreviations**: CI = confidence interval; USD = U.S. dollars.

* Unweighted number of persons who received positive or negative SARS-CoV-2 test results.

**^† ^**p-value for weighted Wald chi-square test; all p-values <0.05 indicate significant differences.

**^§ ^**Other marital status includes separated, divorced, and widowed.

^¶ ^Employed includes full-time or part-time work and self-employment; unemployed includes students, homemakers, and those who were not employed currently or were unable to work.

** Other race/ethnicity includes Native American or Alaskan Native, Asian, and other (unspecified).

**^†† ^** *Northeast*: Connecticut, Maine, Massachusetts, New Hampshire, New Jersey, New York, Pennsylvania, Rhode Island, and Vermont; *Midwest*: Illinois, Indiana, Iowa, Kansas, Michigan, Minnesota, Missouri, Nebraska, North Dakota, Ohio, South Dakota, and Wisconsin; *South*: Alabama, Arkansas, Delaware, District of Columbia, Florida, Georgia, Kentucky, Louisiana, Maryland, Mississippi, North Carolina, Oklahoma, South Carolina, Tennessee, Texas, Virginia, and West Virginia; *West*: Alaska, Arizona, California, Colorado, Hawaii, Idaho, Montana, Nevada, New Mexico, Oregon, Utah, Washington, and Wyoming.

Overall, 603 (86.5%) respondents who received a positive test result and 1,526 (61.7%) of those who received a negative test result reported any initial symptoms. Among respondents who reported an initial symptom, more respondents who received a positive test result (76.2%) than those who received a negative test result (69.6%) reported persistence (>4 weeks) of at least one symptom (p = 0.005) (Supplementary Table, https://stacks.cdc.gov/view/cdc/108815). Hair loss (58.3%), cognitive dysfunction*** (55.5%), shortness of breath (52.8%), and postexertional malaise^†††^ (49.6%) persisted for ≥50% of those who received a positive test result and initially reported these symptoms; other symptoms, such as fatigue (48.4%), change in smell or taste (46.4%), cough (36.2%), and headache (31.1%) persisted for <50%.

A higher proportion of respondents who received a positive test result than those who received a negative test result reported any long-term symptoms (65.9% versus 42.9%; p<0.05) (Table 2). The most common symptoms were fatigue (22.5% versus 12.0%), change in smell or taste (17.3% versus 1.7%), shortness of breath (15.5% versus 5.2%), cough (14.5% versus 4.9%), and headache (13.8% versus 9.9%). Among only respondents who reported any long-term symptoms, the most common symptoms among those who received a positive test result compared with those who received a negative test result were fatigue (34.2% versus 28.0%), change in smell or taste (26.2% versus 3.9%), shortness of breath (23.6% versus 12.1%), cough (22.0% versus 11.5%), and headache (20.9% versus 23.0%) (all p<0.05 except for headache).

**TABLE 2:** Prevalence of symptoms lasting >4 weeks among respondents aged ≥18 years who received at least one positive SARS-CoV-2 test result or only negative SARS-CoV-2 test results since January 2020 — Porter Novelli Internet survey, United States, April 2021

Symptom	SARS-CoV-2 test result, weighted % (95% CI)			
	Symptom prevalence among all persons receiving testing		Symptom prevalence among persons receiving testing who reported a symptom lasting >4 weeks since onset	
	Respondents who received a positive test result (n = 698)*	Respondents who received a negative test result (n = 2,437)*	Respondents who received a positive test result (n = 465)*	Respondents who received a negative test result (n = 1,058)*
**Any symptom**	**65.9^†^ (61.9–69.8)**	**42.9 (40.8–45.1)**	**—**	**—**
1 symptom only	27.2^†^ (23.7–30.8)	18.7 (17.0–20.4)	41.4 (36.5–46.3)	43.6 (40.3–46.9)
2 symptoms	14.0^†^ (11.1–16.8)	9.5 (8.2–10.7)	21.2 (17.1–25.3)	22.1 (19.3–24.8)
≥3 symptoms	24.7^†^ (21.0–28.3)	14.7 (13.2–16.3)	37.4 (32.5–42.4)	34.3 (31.1–37.5)
Fatigue/Tired/Weakness	22.5^†^ (19.0–26.1)	12.0 (10.6–13.4)	34.2^†^ (29.3–39.1)	28.0 (25.0–31.0)
Change in smell or taste	17.3^†^ (14.1–20.4)	1.7 (1.1–2.3)	26.2^†^ (21.8–30.7)	3.9 (2.6–5.3)
Shortness of breath or breathlessness	15.5^†^ (12.4–18.7)	5.2 (4.2–6.2)	23.6^†^ (19.1–28.1)	12.1 (9.9–14.2)
Cough	14.5^†^ (11.6–17.4)	4.9 (4.0–5.9)	22.0^†^ (17.8–26.2)	11.5 (9.4–13.6)
Headache	13.8^†^ (10.9–16.7)	9.9 (8.6–11.2)	20.9 (16.7–25.1)	23.0 (20.2–25.8)
Problems sleeping	12.0^†^ (9.3–14.7)	16.5 (14.8–18.1)	18.1^†^ (14.2–22.1)	38.3 (35.1–41.6)
Joint or muscle pain	11.1 (8.4–13.9)	12.4 (10.9–13.9)	16.9^†^ (12.9–20.9)	28.9 (25.8–32.0)
Cognitive dysfunction^§^	10.2^†^ (7.7–12.8)	7.3 (6.1–8.4)	15.5 (11.8–19.3)	16.9 (14.4–19.4)
Chest pain or pressure	7.3^†^ (5.2–9.4)	2.3 (1.6–2.9)	11.0^†^ (7.9–14.2)	5.3 (3.7–6.8)
Change in mood	6.6 (4.6–8.7)	8.8 (7.6–10.0)	10.1^†^ (7.1–13.1)	20.6 (17.9–23.2)
Postexertional malaise^¶^	6.1^†^ (4.1–8.0)	2.4 (1.7–3.0)	9.2^†^ (6.3–12.2)	5.5 (3.9–7.0)
Stomach pain	5.8 (3.9–7.7)	5.1 (4.1–6.1)	8.9 (6.0–11.7)	11.9 (9.7–14.1)
Hair loss	5.6 (3.7–7.5)	4.1 (3.3–5.0)	8.5 (5.6–11.3)	9.7 (7.6–11.7)
Diarrhea	5.3 (3.3–7.2)	3.3 (2.6–4.1)	8.0 (5.0–10.9)	7.8 (6.0–9.5)
Sore throat	4.9^†^ (3.1–6.8)	1.7 (1.1–2.2)	7.5^†^ (4.7–10.3)	3.9 (2.7–5.1)
Fever or chills	4.9^†^ (3.0–6.8)	1.9 (1.4–2.5)	7.5 (4.7–10.3)	4.5 (3.2–5.8)
Palpitations (heart racing or pounding)	4.5 (2.7–6.3)	2.5 (1.9–3.2)	6.8 (4.1–9.5)	5.9 (4.3–7.5)
Nausea/Vomiting	4.1^†^ (2.5–5.8)	1.9 (1.3–2.4)	6.3 (3.8–8.8)	4.3 (3.0–5.7)
Other symptom	1.3 (0.3–2.2)**	1.0 (0.6–1.5)	2.0 (0.5–3.4)**	2.4 (1.4–3.4)

**Abbreviation:** CI = confidence interval.

* Unweighted number of persons who received at least one positive or only negative SARS-CoV-2 test results.

^† ^p-value for weighted Wald chi-square test <0.05, indicating significant differences between those receiving a positive test result and those receiving a negative SARS-CoV-2 test result.

^§^ Difficulty thinking clearly, concentrating, forgetfulness, memory loss, or “brain fog.”

^¶^ Worsening of symptoms after even minor physical, mental, or emotional exertion.

** Estimate is unstable; relative standard error is >30%.

A larger proportion of respondents who received a positive test result than those who received a negative test result reported seeing a health care professional (54.1% versus 42.5%; p<0.001) or going to urgent or emergency care (19.5% versus 14.0%, p = 0.008) for symptoms when they first occurred; rates of hospitalization were similar (10.4% versus 9.3%) (Table 3). Among those reporting any long-term symptoms, fewer respondents who received a positive test result than those who received a negative test result reported seeing a health care professional for long-term symptoms at least once (24.7% versus 35.8%) or more than once (17.6% versus 27.9%).

**TABLE 3:** Frequency of health care use, vaccination, and reported vaccine effects on symptoms lasting >4 weeks among respondents aged ≥18 years who received at least one positive SARS-CoV-2 test result or only negative SARS-CoV-2 test results since January 2020 — Porter Novelli Internet survey, United States, April 2021

Item	Weighted % (95% CI)		p-value*
	At least one positive SARS-CoV-2 test result	All negative SARS-CoV-2 test results	
**Health care utilization among those with any initial symptom **	603^†^	1,526^†^	N/A
Saw health care professional for symptoms	54.1 (49.7–58.6)	42.5 (39.8–45.2)	<0.001
Went to urgent or emergency care for symptoms	19.5 (16.0–23.0)	14.0 (12.1–15.9)	0.008
Hospitalized for symptoms	10.4 (7.8–13.0)	9.3 (7.6–11.0)	0.492
**Health care utilization among those with symptoms lasting >4 wks (long-term symptoms) **	465^†^	1,058^†^	N/A
Saw health care professional for long-term symptoms	24.7 (20.5–28.9)	35.8 (32.6–39.0)	<0.001
Saw health care professional more than once for long-term symptoms	17.6 (13.7–21.5)	27.9 (24.8–30.9)	<0.001
**COVID-19 vaccination status**	698^†^	2,437^†^	N/A
Received at least 1 dose of vaccine^§^	28.3 (24.5–32.0)	39.4 (37.3–41.5)	<0.001
Having long-term symptoms was a motivator to receive or consider receiving vaccine	11.0 (8.0–14.0)	7.0 (5.3–8.7)	0.023
**Reported vaccination effects on long-term symptoms^¶^**	100^†^	285†	N/A
Vaccine made symptoms better**	28.7 (18.6–38.7)	15.7 (11.3–20.0)	0.023
Vaccine did not affect symptoms at all	26.4 (16.7–36.0)	59.2 (53.1–65.4)	<0.001
Vaccine made symptoms worse^††^	16.1 (8.4–23.7)	11.2 (6.9–15.4)	0.271
Symptoms were gone before receiving vaccine	28.4 (18.4–38.5)	13.1 (8.9–17.3)	0.007

**Abbreviations**: CI = confidence interval; N/A = not applicable.

* p-value for weighted Wald chi-square test; all p-values <0.05 indicate significant differences.

^†^ Unweighted number of persons who received positive or negative SARS-CoV-2 test results.

**^§ ^**189 of 698 respondents who received a positive test result and 961 of 2,437 respondents who received a negative test result reported receiving 1 dose of vaccine.

**^¶ ^**Respondents who ever experienced a long-term symptom and received at least 1 vaccine dose.

** Includes those reporting receiving a vaccine made symptoms a lot better, somewhat better, or a little better.

**^†† ^**Includes those reporting receiving a vaccine made symptoms a little worse, somewhat worse, or a lot worse.

Fewer respondents who received a positive test result than those who received a negative test result reported receiving ≥1 dose of a COVID-19 vaccine (28.3% versus 39.4%). Among those who ever experienced any long-term symptoms, more respondents who received a positive test result than those who received a negative test result reported that having long-term symptoms motivated them to receive or consider receiving a COVID-19 vaccine (11.0% versus 7.0%) and believed that receiving the vaccine made their long-term symptoms better (28.7% versus 15.7%; p = 0.023), or that their symptoms were gone before receiving the vaccine (28.4% versus 13.1%). A similar percentage of respondents who received a positive test result (16.1%) and those who received a negative test result (11.2%) reported that receiving the vaccine made their long-term symptoms worse (p = 0.271), whereas 26.4% of respondents who received a positive test result and 59.2% of those who received a negative test result believed that receiving a vaccine did not affect their symptoms (p<0.001).

## Discussion

In this convenience sample of U.S. adults, the prevalence of long-term symptoms often associated with SARS-CoV-2 infection was higher among respondents who ever received a positive test result than among those who always received a negative test result, and symptoms in these persons tended to persist for >4 weeks. Previous studies have found that nonhospitalized persons with SARS-CoV-2 infection have higher prevalence of some long-term symptoms or conditions than nonhospitalized persons with negative SARS-CoV-2 test results (*2*–*5*). Similarly, in this investigation, more respondents who received a positive test result (65.9%) than those who received a negative test result (42.9%) experienced any long-term symptoms, and approximately one half of these symptoms were more likely to be reported among those who received a positive test result.

Early data on post-COVID conditions primarily came from hospitalized cohorts (*1*,*6*); more recent reports describe post-COVID conditions among nonhospitalized, asymptomatic, or mildly ill patients (*1*,*7*). The prevalence of the most common long-term symptoms among respondents who received a positive test result in this investigation was similar to that in earlier studies (*1*,*8*). Many studies on post-COVID conditions lack comparisons with the general population of adults with negative test results for SARS-CoV-2; however, this investigation included a comparison group, allowing for assessment of background symptom frequencies. Estimating population-level frequency of specific long-term symptoms among the general population and patients infected with SARS-CoV-2 could help health care professionals better understand the types and prevalences of symptoms their patients might experience and could help guide health systems in preparing care management strategies for patients with post-COVID conditions.

Among respondents who initially reported symptoms during the month of their first positive test results, >75% reported persistence of any symptoms >4 weeks, with hair loss, cognitive dysfunction, shortness of breath, and postexertional malaise persisting in approximately one half of respondents. This finding is consistent with findings from other studies reported in a systematic review (*1*) and provides patient-level perspective on long-term symptoms associated with COVID-19; taken together, these studies highlight the importance of continued monitoring and clinical care for long-term symptoms among patients who have these symptoms early in the course of their illness.

With the increasing availability of COVID-19 vaccines, how vaccination affects post-COVID conditions remains unclear. Compared with respondents who received a negative test result, a higher proportion of those who received a positive test result believed that receiving a COVID-19 vaccine made their long-term symptoms better, and no difference was found in reported beliefs that receiving a vaccine made long-term symptoms worse. Early findings indicate that vaccination is not associated with worsening of post-COVID conditions^§§§^ (*9*). However, because no data were collected on the trajectory of long-term symptoms in persons who had not been vaccinated, whether any of the observed changes in symptoms are attributable to vaccination is uncertain. More data are needed to fully understand the effects of COVID-19 vaccines on persons with post-COVID conditions.

The findings in this report are subject to at least six limitations. First, the study used a nonprobability-based sample, which limits its generalizability. Second, responses were self-reported and thus subject to recall bias. Third, new symptoms occurring after the month when the first positive COVID-19 test result was received among those who received a positive test result were not assessed, and the reported symptoms could not be linked directly to SARS-CoV-2. Fourth, because the survey did not ask about symptom duration or severity, differences in duration or severity of long-term symptoms in respondents who received a positive rather than a negative test result could not be assessed. Fifth, respondents who always received a negative test result generally had a longer period in which to report symptoms, potentially inflating prevalence of their health care use and long-term symptoms. Finally, this study could not assess validity of SARS-CoV-2 tests, and some false-positive or false-negative test results might have resulted in misclassification of some respondents.

These findings can help guide public health preparedness efforts, resource needs for care and management of persons with post-COVID conditions, and communication about experiences with vaccination. The findings can also aid efforts to address post-COVID conditions, including helping health care professionals recognize the most common symptoms and optimize care for patients whose symptoms persist. Future research to assess long-term symptoms and risk factors, including disease severity, disease duration, and sociodemographic characteristics, will be important to help guide current and future health care services.

**Summary**

**What is already known about this topic?**

Long-term symptoms associated with COVID-19 represent an emerging public health concern.

**What is added by this report?**

In a nonprobability-based sample of U.S. adults tested for SARS-CoV-2, symptoms often associated with SARS-CoV-2 infection were common; 65.9% of respondents whose SARS-CoV-2 test results were positive reported symptoms lasting >4 weeks compared with 42.9% of those whose test results were negative. More persons who received positive test results (76.2%) reported persistence (>4 weeks) of at least one initially occurring symptom compared with those whose test results were negative (69.6%).

**What are the implications for public health practice?**

These findings can aid efforts to address post-COVID conditions and messaging on potential benefits of vaccination.

## Footnotes

*
https://styles.porternovelli.com/pn-view-panels/

†
https://engine-insights.com/product/caravan/

§
https://luc.id/marketplace/

¶ Change in mood; change in smell or taste; chest pain or pressure; cough; diarrhea; difficulty thinking clearly, concentrating, forgetfulness, memory loss, or “brain fog”; fatigue, tired, or weakness; fever or chills; hair loss; headache; joint or muscle pain; nausea or vomiting; palpitations (heart racing or pounding); postexertional malaise (worsening of symptoms after even minor physical, mental, or emotional exertion); problems sleeping; shortness of breath or breathlessness; sore throat; stomach pain; or other symptom.

** Among those who experienced long-term symptoms and reported receiving ≥1 dose of a COVID-19 vaccine (100 respondents who received a positive test result and 285 respondents who received a negative test result).

†† Respondents were instructed, “Please select only one answer and do not count side effects (symptoms that you had in the first 7 days after getting a COVID-19 vaccine).” Responses included, “Made my symptoms a lot better,” “Made my symptoms somewhat better,” “Made my symptoms a little better,” “Did not affect my symptoms at all,” “Made my symptoms a little worse,” “Made my symptoms somewhat worse,” “Made my symptoms a lot worse,” “Made some of my symptoms better and some worse,” and “Not applicable: My symptoms were gone before I got the vaccine.” Because of small cell sizes, those reporting that receiving a vaccine made their symptoms a lot, somewhat, or a little better were grouped under “Getting vaccine made symptoms better” and those reporting that receiving vaccine made their symptoms a lot, somewhat, or a little worse were grouped under “Getting vaccine made symptoms worse.”

§§ Urban, suburban, or rural.

¶¶ 45 C.F.R. part 46; 21 C.F.R. part 56; 42 U.S.C. Sect. 241(d); 5 U.S.C. Sect. 552a; 44 U.S.C. Sect. 3501 et seq.

*** Cognitive dysfunction refers to difficulty thinking clearly, concentrating, forgetfulness, memory loss, or “brain fog.”

††† Worsening of symptoms after even minor physical, mental, or emotional exertion.

§§§
https://www.medrxiv.org/content/10.1101/2021.03.11.21253225v2
